# Depressive symptoms are associated with social isolation in face-to-face interaction networks

**DOI:** 10.1038/s41598-020-58297-9

**Published:** 2020-01-29

**Authors:** Timon Elmer, Christoph Stadtfeld

**Affiliations:** Social Networks Lab, Department of Humanities, Social and Political Sciences, ETH Zürich, Zürich, Switzerland

**Keywords:** Psychology, Human behaviour

## Abstract

Individuals with depressive symptoms are more likely to be isolated in their social networks, which can further increase their symptoms. Although social interactions are an important aspect of individuals’ social lives, little is known about how depressive symptoms affect behavioral patterns in social interaction networks. This article analyzes the effect of depressive symptoms on social interactions in two empirical settings (N_total_ = 123, N_dyadic relations_ = 2,454) of students spending a weekend together in a remote camp house. We measured social interactions between participants with Radio Frequency Identification (RFID) nametags. Prior to the weekend, participants were surveyed on their depressive symptoms and friendship ties. Using state-of-the-art social network analysis methods, we test four preregistered hypotheses. Our results indicate that depressive symptoms are associated with (1) spending less time in social interaction, (2) spending time with similarly depressed others, (3) spending time in pair-wise interactions rather than group interactions but not with (4) spending relatively less time with friends. By “zooming in” on face-to-face social interaction networks, these findings offer new insights into the social consequences of depressive symptoms.

## Introduction

Social interactions are the smallest building blocks of interpersonal social networks and are a prerequisite of the formation of functional social relationships. The lack of social interactions and social relationships (i.e., social isolation) can have detrimental effects on an individual’s physical and psychological health. Social isolation increases the risk for coronary heart disease, stroke, and mortality^1–3^ and can negatively influence psychological health leading to depressive symptoms^4,5^.

But social isolation can also be the *consequence* of depressive symptoms. It is well established that individuals with depressive symptoms have less rewarding and more dysfunctional social relationships^6–8^. In that vein, longitudinal social network studies have shown that depressive symptoms affect the creation, maintenance, and termination of social ties^9,10^. While the effects of depressive symptoms have mostly been examined in self-reported friendship networks, many processes are in fact argued to operate on the more fine-grained level of social interactions^9,11–14^. Investigating the social processes on an interaction level can help us to understand how depressive symptoms contribute to being socially isolated. This paper thus develops and tests four preregistered hypotheses on how depressive symptoms affect face-to- face interactions in social networks.

The first hypothesis (*depression-isolation hypothesis)* states that depressive symptoms are associated with less social interactions. It has been argued that depressive symptoms are accompanied by a change of social skills and motivation to socialize (e.g., more reassurance seeking)^7,15,16^. Individuals with more depressive symptoms may experience fewer social interactions because: (1) they may elicit rejection from others as they induce a negative mood in their interaction partners^17–19^ and (2) they are likely to receive less reinforcement from the social environment, which contributes to a feeling of discomfort in social interactions and decreased social participation^7,20,21^. In line with these theoretical considerations, Brown and colleagues^20^ have reported a negative association between depressive symptoms and the amount of self-reported social interactions. At the same time, other studies reported no differences in the quantity of social interactions but only on qualitative aspects of social interactions^22–24^. These self-report-based findings, however, may entail measurement biases that are associated with how depressed individuals self-report social interactions (e.g., having more negative social self-perceptions)^25,26^. The use of a direct behavioral measure of social interactions that we propose in this article allows us to overcome these measurement biases.

The second hypothesis (*depression-homophily hypothesis*) states that individuals are more likely to interact with others who have a similar level of depression^9,10^. The tendency to bond with similar others (homophily)^27^ has been found to be one of the most consistent patterns in social networks. It is expected to be prevalent on the depression scale, as sharing emotional states with similar others can lead to more compassion and self-disclosure and thus to more rewarding interactions^28^.

The third hypothesis (*depression-friendship hypothesis*) states that individuals’ depressive symptoms are associated with the relative time that they interact with friends. The direction of this association, however, is unclear. We assume that friends tend to spend time together^29^ and that they will be more aware of each other’s mental health than non-friends (e.g., through signs of verbal or non-verbal behaviors in previous interactions)^7^. On the one hand, some evidence suggests that friends are less rejecting of individuals with depressive symptoms than strangers^30^. This would indicate a positive association. On the other hand, individuals with more depressive symptoms are more likely to interact with others in a way that focuses on their problems, seeking reassurance, and to bring others to solve their problems^16^. This tendency might be particularly noticeable when depressed individuals interact with their friends as these relations are more characterized by self-disclosure^31,32^. This tendency may lead friends to avoid social interactions with individuals with more depressive symptoms. In one empirical study, Brown *et al*.^20^ showed that depressed individuals tend to interact with their friends less often, compared to healthy controls.

The fourth hypothesis (*dyadic-isolation hypothesis*) states that individuals’ depressive symptoms are associated with a higher number of interactions in pairs (dyads), rather than interacting in groups of three or more. Depressed individuals may show a higher frequency of dyadic interaction because of their tendency of “discussing and revisiting problems, speculating about problems, and focusing on negative feelings” (p. 1830) in dyadic social interactions that are characterized by more self-disclosure (i.e., co-rumination)^33^. If co-rumination is more likely to occur in pairs, this could lead to an over-representation of dyadic interactions among depressed individuals.

**Figure 1:**
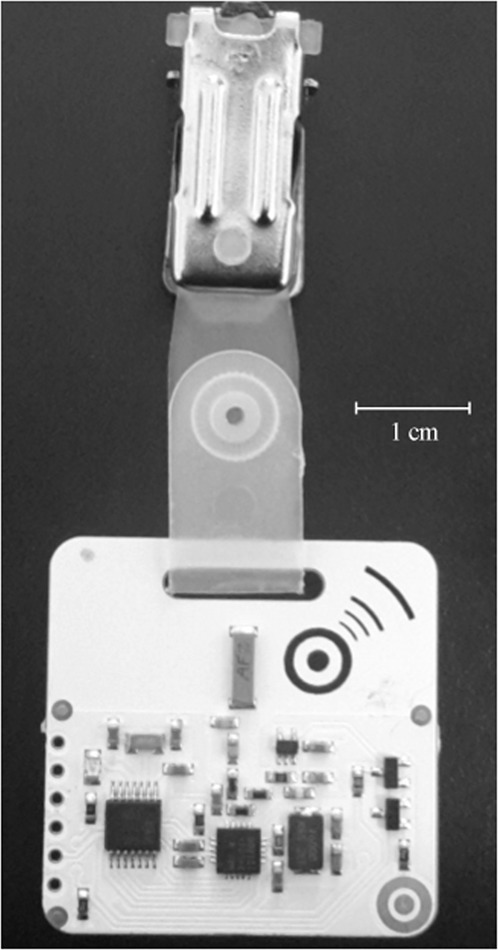
A picture of an RFID badge.

The present study is situated in a context in which individuals (first-week undergraduate students) get to know each other in the process of an emerging social group. Two independent cohorts participated in this study (N_1_ = 73, N_2_ = 50). About 22% of the participants reported clinically relevant levels of depressive symptoms (more than 16 scale points) and 39% reported sub-clinical levels of depressive symptoms (between 9 and 16 scale points)^34^. Rather than using self-reports of social interactions, we collected fine-grained data on face-to-face interactions using newly developed Radio Frequency Identification Devices (RFID)^35^. Figure 1 shows a picture of an RFID badge, which is usually worn as part of a nametag. The badge automatically records when study participants face each other frontally in very close proximity that is typically associated with a social interaction. Recently, RFID badges have been validated for measuring such face-to-face social interaction^36^. The data collected by the RFID badge are combined with self-reported data on friendship relations and depressive symptoms assessed prior to the social interactions. We apply state-of-the-art statistical methods of social network analysis^37,38^ that take into account that relational observations are not statistically independent. We, thereby, test four preregistered hypotheses (osf.io/xce9g) on the interplay between social interactions and depressive symptoms, while taking the role of preexisting friendship into account. Furthermore, we statistically control for the effects that the Big Five personality traits have on social interaction, as they are argued to affect these^39^. The unique social network design allows us to test these relation hypotheses that require data of a closed group of interacting individuals and data on the depressive symptoms of (possibly) all individuals in the group.

Social isolation can be cause and consequence of depressive symptoms, potentially trapping some individuals in a vicious cycle. Understanding the fine-grained interaction patterns of individuals with depressive symptoms can be a first step towards future interventions to break this vicious cycle of social isolation and depressive symptoms.

## Results

### Description of the data

**Figure 2:**
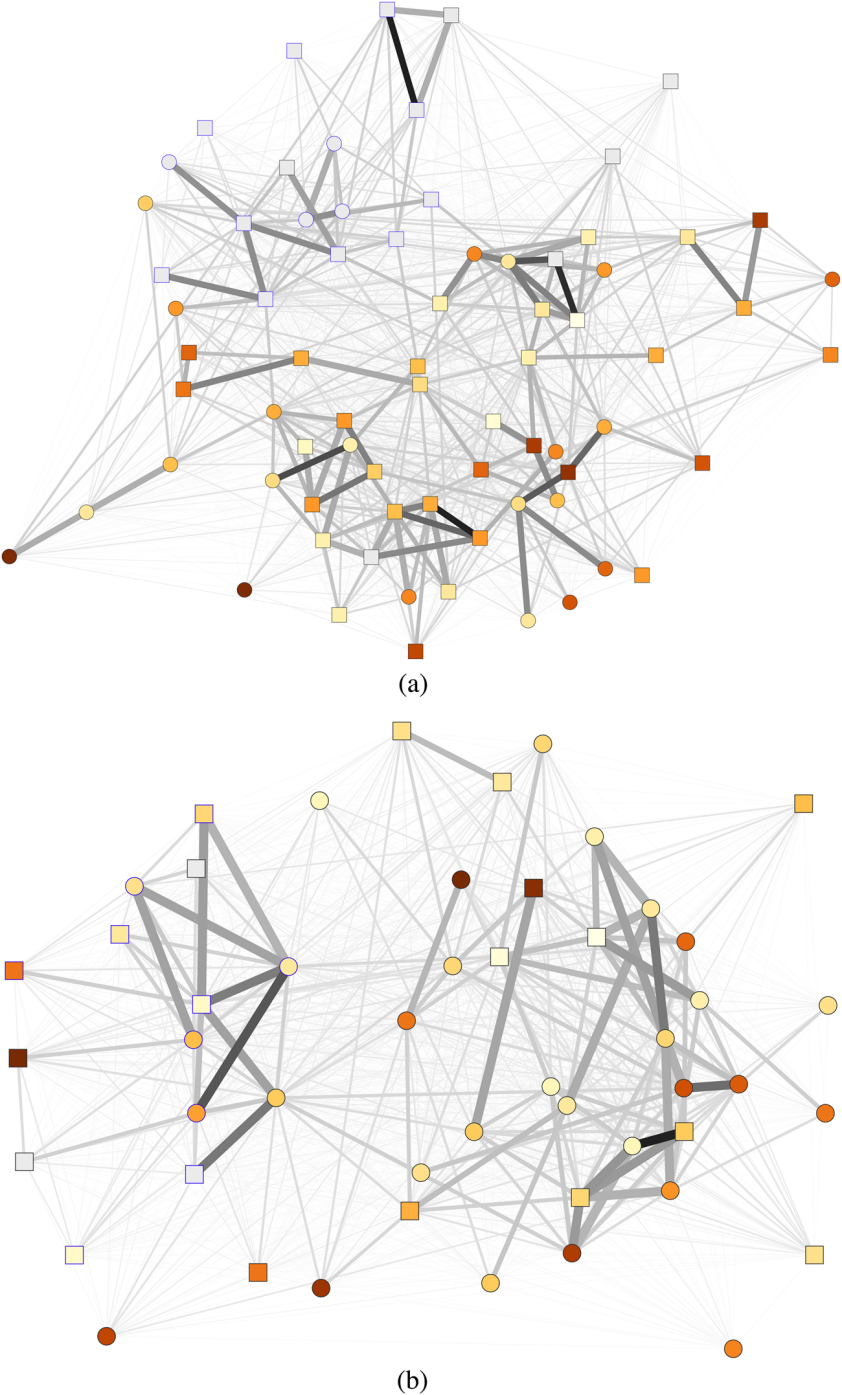
Durations of social interactions over the course of the data collection for sample one (**a**) and sample two (**b**); tie color and width = interaction duration, blue node frame = student organization member, color = depressive symptoms (dark red = high, yellow = low, grey = missing value), circles = females, squares = males, plotted with visone^43^.

On average, individuals reported a depression score of 10.28 (*SD* = 5.25) in sample one and 11.98 (*SD* = 7.97) in sample two. According to the screening criteria defined by Radloff^40^, 15% of the respondents in sample one and 29% of sample two show clinically relevant levels of depressive symptoms. In representative samples of university students, similar prevalences have been measured^41,42^. In sample one, we also collected data of individuals that were in the same study group but chose not to attend this voluntary social event or signed up after all slots have been taken. Those individuals attending the weekend did not differ in their level depressive symptoms from those that did not attend this voluntary event (N = 119), t(174) = 0.15, p = 0.881. A total number of 23,452 social interaction events were recorded in sample one and 12,225 in sample two. These numbers relate to the raw data of recorded RFID interactions over the whole weekend. The average duration of interactions was 94.51 (*SD* = 212.77) seconds and 86.81 (*SD* = 186.32) seconds, respectively. The large standard deviation indicates the amount of variability between pairs of students. These social interactions were aggregated to one adjacency matrix per sample where each entry represents the total duration of social interactions between individual *i* and *j*. Each participant on average interacted 16.87 hours (*SD* = 7.27) with others in sample one and 11.79 hours (*SD* = 6.41) in sample two. Figure 2 shows these interaction networks. Each individual is represented as a node, where the node color indicates the degree of depressive symptoms (dark red = high, yellow = low, grey = missing value). The thickness of ties denotes how long two individuals have interacted with each other. The networks exhibit typical social network structures — for example, one can see that interactions tend to cluster within certain regions of the network.

On average, the participants reported 0.66 friendship ties (*SD* = 1.28) in sample one and 2.14 (*SD* = 2.13) in sample two. Because the participants of sample two knew each other for a week longer, more friendship relations were established. In total, 48 ties (sample one) and 107 ties (sample two) were reported. Of those 20 were mutual and 28 were asymmetric in sample one. In sample two, 78 friendship ties were mutual and 29 were asymmetric.

Before testing our hypotheses with multivariate social network methods, we – in the next paragraph – show how depressive symptoms and different aspects of social interactions correlate bivariately on the individual level. Table 1 shows the correlation coefficients of depressive symptoms with properties of the interaction network. These coefficients show that depressive symptoms are negatively correlated with how much time individuals spend in social interactions. Depressive symptoms do not correlate with the amount of time spent with friends (symmetrized measure). However, there is a negative correlation with the amount of time spent with mutual friends. We find no evidence for a correlation between depressive symptoms and the amount of time spent in dyadic interactions, but a negative correlation with the amount of time spent in group interactions. These differences between dyadic and group interactions are also reflected in the positive correlation of depressive symptoms with one’s ratio of dyadic interactions in all social interactions.

**Table 1:** Pearson correlations between depressive symptoms and interaction aggregates.

	(1)	(2)	(3)	(4)	(5)	(6)	(7)	(8)	(9)
Depression (1)									
Age (2)	−0.13								
Gender (1 = female)^a^ (3)	0.17	−0.11							
T in interaction (4)	−0.23*	0.08	0.04						
T per friend (5)	−0.07	−0.11	−0.03	0.44***					
T per mutual friend (6)	−0.30*	0.09	0.00	0.38**	0.91***				
T per asymmetric friend (7)	−0.08	−0.11	−0.05	0.44***	0.99***	0.91***			
T in dyadic interactions (8)	−0.08	0.12	0.14	0.76***	0.22*	0.28*	0.22*		
T in group interactions (9)	−0.26**	0.03	−0.01	0.91***	0.46***	0.35**	0.46***	0.42***	
Ratio dyadic interactions (10)	0.26**	0.00	0.07	−0.53***	−0.38***	−0.22	−0.39***	0.09	−0.80***

Note. *p < 0.05, **p < 0.01, ***p < 0.001 (two-sided p-values). T = time [in sec] normalized by the hour (so that the two samples are comparable). ^a^Spearman’s rank correlations.

### Multi-group MRQAPs

To test the multivariate relationships between social interactions and individual’s attributes, we conducted a multi-group MRQAP analysis^38^. Parameters of a MRQAP can be interpreted exactly like parameters of a linear regression model, but because the assumption of independent observations is violated, MRQAPs rely on a permutation-based test to obtain statistical inference (more details on MRQAPs can be found in the methods section). The result of our MRQAP analysis is shown in Table 2, reporting the estimates of observed network $\hat{\beta }$ and the comparison with the *β* estimated under 5,000 network permutations. The mean value of the estimate under the permuted dependent networks is indicated by $E(\beta )$.

**Table 2:** Multi-group QAP results on log-transformed interaction durations of dyads.

	Estimate	*p*	E(Est.)	Percentiles	
				2.5th	97.5th
Intercept	2.504**	0.005	1.820	1.290	2.346
Sample two	0.806	0.344	0.835	0.693	0.974
At least one female	−0.095	0.129	−0.003	−0.160	0.155
Both female	−0.148*	0.036	0.004	−0.158	0.160
Age mean (centered)	0.065*	0.013	0.000	−0.059	0.057
Age similarity	0.042**	0.009	0.000	−0.035	0.035
One student organization	−0.028	0.450	−0.001	−0.452	0.451
Same student status	0.269	0.115	−0.003	−0.456	0.435
Being friends	2.128***	<0.001	0.007	−0.453	0.477
Depression mean	−0.059***	<0.001	0.000	−0.023	0.024
Depression similarity	0.047**	0.004	0.000	−0.035	0.034
Depression mean * depression similarity	−0.004***	0.001	0.000	−0.002	0.002
Depression mean * being friends	−0.012	0.333	0.000	−0.053	0.052
R^2^	0.123				
Adj. R^2^	0.119				

Note. Multigroup MRQAPs with 5,000 Y-permuted samples. *p < 0.05, **p < 0.01, ***p < 0.001 (two-sided p-values). We report p-values because confidence intervals cannot be computed for MRQAPs. The percentiles describe the distribution under the null hypothesis and can be interpreted similarly to confidence intervals. Various robustness analyses (the two samples separately, a standard linear regression, with a non-log-transformed dependent matrix, with non-merged RFID data, and including Big Five personality traits) are reported in Table S1 and Table S2 of the Supplementary Materials.

The results of the multi-group MRQAPs support the notion of *depression isolation*; dyads with a high mean in depressive symptoms were less likely to interact. It has to be noted that the effect size of the estimate cannot be interpreted directly due to the log transformation of the dependent matrix. The following example should illustrate the size of this effect: The interaction time between two individuals with a depression score of 5 each is estimated to be 9.12 seconds per hour (exp(2.504-0.059*5)), whereas an interaction between two individuals with a depression score of 20 is estimated to last for only 3.76 seconds per hour (exp(2.504-0.059*20); considering that everything else is the reference category - for instance, that there is no friendship tie present).

There was a positive effect for depression similarity; this suggests that social interactions were more likely between individuals that reported a similar level of depressive symptoms (*depression-homophily* hypothesis). Moreover, the interaction between depression mean and depression similarity was a negative predictor of social interactions, showing that depression homophily is stronger at the lower (the less depressed) end of the scale.

**Figure 3:**
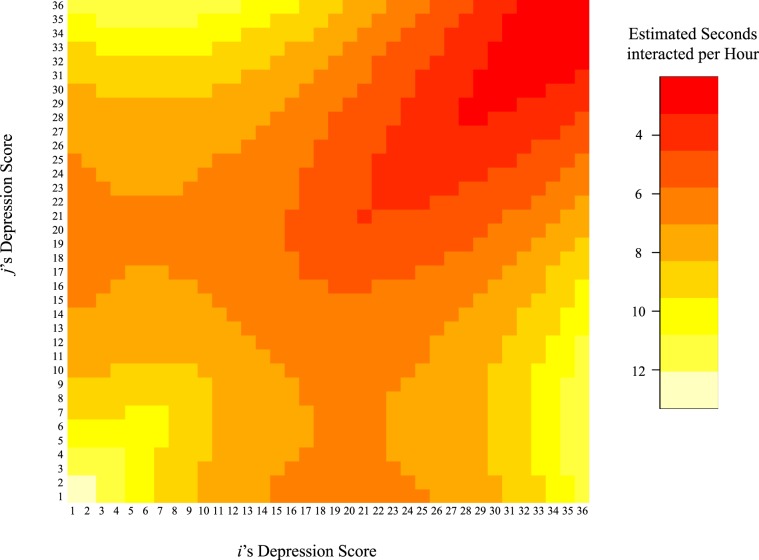
${\hat{y}}_{ij}$**(**in sec/h) for depression values between 1 and 36 (i.e., the range of observed values) for the case of all reference categories (of e.g., gender, age, friendship ties).

The multivariate interplay between predictors of social interactions and their effect size can be shown with a *selection table* where the estimates of a multivariate analysis are used to calculate an estimate for the dependent variable (i.e., social interaction duration) for various configurations of the predictors^9^. In our case, we want to show how various levels of depressive symptoms of individual *i* and *j* predict social interaction duration of the dyad *y*_*ij*_ with the estimates of depression mean, depression similarity, and their interaction. The values for ${\hat{y}}_{ij}$ of the observed range of depressive symptoms of *i* and *j* (0 to 36) are shown in a heatmap in Fig. 3. Details on the computation of ${\hat{y}}_{ij}$ for this Figure can be found in the Supplementary Material (Section Computation of the Selection Table). For the case of two male students of sample one that are not friends and have the same age (i.e., all reference categories), Fig. 3 shows that interactions where both individuals were highly depressed were the least likely and those most likely were interactions between low depressed individual or when one individual was highly depressed and the other one low in depression.

To investigate how depressive symptoms are associated with the extent to which individuals interact with friends, we tested an interaction of depression mean with the symmetrized friendship matrix. There was no significant effect of depression mean with being friends in predicting social interactions (*depression-friendship hypothesis*). As noted earlier, friendship relations might be mutual or asymmetric (either both individuals consider the relationship as a friendship or just one of the two). Neglecting this information might diffuse the differentiation between weak and strong friendship ties. For this reason, we conducted additional analyses in which we included two matrices capturing the mutual and asymmetric friendship relations instead of one symmetrized friendship matrix.

In those analyses, we find a negative interaction effect of depression mean with being mutual friends in predicting social interactions (β = −0.084, p = 0.029), indicating that depressed individuals tend to interact less with their reciprocated friends than non-depressed individuals. Interestingly, the interaction of asymmetric friendship ties with depression mean was positive but did not predict interaction duration significantly (β = 0.069, p = 0.095). Details on these results are provided in Table S3 of the Supplementary Materials.

Beyond these depression-related findings, the multi-group MRQAP analysis shows significantly higher estimates for sample two and the estimates increased with an increasing mean age and increasing age similarity. Negative estimates were found for both individuals being female, indicating that interactions between two females are less observed than between two males. The overall explained variance of the model is R^2^ = 0.12, which is not very high, but considerable given the large set of factors that potentially affect the formation of social interactions between two individuals.

We conducted a number of robustness analyses of these multi-group MRQAP analyses: (1) for the two samples separately, with a (2) non log-transformed dependent matrix, and (3) with non-merged RFID data (interactions of dyads that were no longer than 75 seconds apart have been merged as recommended by Elmer *et al*.^36^ for improved validity). Also, we included measures of the Big Five personality traits into the model. The results of these robustness analyses can be found in Table S1 and Table S2 of the Supplementary Material. All these robustness analyses yield that the findings of this study are robust against different data treatments, within each sample, and when controlling for the effect of personality traits. The exception being the depression similarity effect, which is not a significant predictor in the separate analysis of sample two (β = 0.024, p = 0.142) and when modeling the non log-transformed duration matrix (β = 0.557, p = 0.213), and the depression mean effect which is not significant when modeling the non log-transformed duration matrix (β = −0.474, p = 0.160).

### Dyadic and group interactions

Finally, we tested the assumption that individuals with more depressive symptoms spend relatively more time in dyadic interactions than in group interactions (*dyadic-isolation hypothesis*). This hypothesis cannot be tested with the MRQAP as the unit of analysis is beyond a dyadic relation. To account for the interdependencies between observations, we performed a permutation-based correlation test of depressive symptoms on the ratio of dyadic interactions in all social interactions. Permuting the dependent variable (i.e., the ratio) here follows the general logic of bivariate QAPs^37^. There was a positive correlation between an individual’s ratio of dyadic interactions and depressive symptoms and (*r*(121) = 0.263, p = 0.003, 5,000 Y-permutations). In other words, the more depressive symptoms an individual reports, the smaller is the proportion of group interactions of the total time spent in social interactions.

## Discussion

In this study, we investigated how individuals’ depressive symptoms affect social interaction networks within two independent student communities spending a weekend socializing in a remote camp house. We find that individuals’ depressive symptoms are associated with spending less time in social interactions. This is in line with our depression-isolation hypothesis. We also find that individuals tend to interact with others that have a similar level of depressive symptoms, as postulated by our depression-homophily hypothesis. This homophily effect is more pronounced on the lower end of the depression scale. We find no support for the depression-friendship hypothesis, stating that individuals’ depressive symptoms are associated with the extent to which they interact with friends. In further explorations, we find that the likelihood of interacting with mutual friends (i.e., both individuals nominating each other) decreases with higher depression scores. We find no such effects for asymmetric friendship ties (i.e., only one friendship nomination). This might indicate that the hypothesized association depends on the strength of a friendship relation. In line with the dyadic-isolation hypothesis, depressive symptoms are associated with the sizes of interaction groups; individuals high in depressive symptoms are more likely to interact in dyads than in groups.

Besides generally lower levels of social interactions, network-specific behavior patterns of individuals with higher levels of depressive symptoms can additionally contribute to their vicious cycle of social isolation and depression. First, the tendency to interact with similarly depressed individuals can lead to more exposure to their dysfunctional attitudes and thus being socially influenced to develop more depressive symptoms^11^. Second, because of the unique support that strong friends can provide (e.g., emotional support), a lack of interactions with those can lead to the development of more symptomology^44^. Third, the tendency of depressed individuals to interact in pairs instead of groups could additionally contribute to the interaction partners’ social isolation, as they are *both* more likely to become dyadically isolated and interact less with other individuals in a group setting.

These findings contribute to the broad literature on the association between depressive symptoms and social interactions. All prior studies rely on self-reports of depression and interaction e.g.,^20,23^. However, more objective measures of social interactions and social network research designs are necessary to explore more complex relational phenomena.

To study the network dimension of social interaction and depressive symptoms, we apply established social network analysis methods (i.e., MRQAP)^38^. These consider that observations were not sampled randomly from a large population (like in most other psychological studies) but consisted of a closed community of individuals where the dependence *between* individual’s depressive symptoms was at the core of the analysis (e.g., how likely is an interaction based on the similarity in depressive symptoms of two individuals). MRQAPs follow the general estimation intuition of a multivariate regression and are thus straightforwardly interpreted as illustrated in our results.

The empirical setting of this study was unique in many ways. First, we measured social interactions with recently developed RFID badges that allowed us to observe individual behavior directly. Given the small number of studies on “actual” behavior, scholars have been encouraged to applying such methods to approach psychological research questions^45^. We deliberately formulated and tested our hypotheses on the interaction level, this way, zooming in on the processes that are usually measured through friendship ties^11–13^. We argue that friendship relations only capture a very specific (and somewhat abstract) form of relations^31^ and thus do not say much about the broad range of social contact individuals have in daily life. Although people tend to interact with their friends frequently, a large proportion of individual’s interactions are with non-friends. Social interactions, on the other hand, are the basic building blocks of social life and also occur frequently with non-friends. Most importantly, social interactions are on the level where social interventions can operate on: Interventions cannot change how many friends one has, but they can change with how many people one socially interacts.

Second, we combined these data with state of the art sociometric data (friendships) and self-report data of depressive symptoms. Thus, applying a multi-method approach, that also allows us to take the association between friendship and social interactions into account.

Third, the fact that the students spent an entire weekend in a remote camp house, constituted an isolated setting where only social interactions between participants were possible. All attendees of the weekends participated in the RFID data collection, providing us with a full-range view on the social interaction dynamics of the participating individuals.

Fourth, we conducted additional analyses in which the effects of the Big Five personality traits on social interactions were statistically controlled for. The findings of this study are robust, even when taking the effects of the Big Five personality traits into account. Hence, depressive symptoms explain unique aspects of social interactions beyond those that can be explained by the Big Five personality traits.

This study also had a number of limitations. First, our empirical setting was in a very specific population and context – a socializing weekend of first semester students. Presumably, all participants felt a norm of being socially engaged at this event. At the same time, friendship relations were often formed relatively recently. Future studies should investigate social interaction networks in different social settings. In that vein, the empirical settings were relatively small. Thus, the application of different types of social interaction measures (e.g., through smartphones^46^) could provide access to broader social settings. Second, in our samples 20% percent of individuals reported depressive symptoms above a clinically relevant cutoff-point. A further extension of this study would be to investigate and replicate the tested hypotheses using a sample where individuals with diagnosed depression are oversampled (e.g., in a psychiatric ward). Nevertheless, given that the social impairment associated with depressive symptoms is argued to increase linearly with the number of symptoms reported^47,48^, our findings potentially provide reliable estimates for social behavior of individuals with depression too. Second, our method of measuring social interaction was limited to assessing quantitative aspects of a social interaction but not qualitative aspects of the social interactions. Hence, we do not know how a potential social skill deficit of depressed individuals actually affected characteristics of social interactions (e.g., eye-contact avoidance of individuals with depression)^7^. Third, we aggregated the social interactions of the two samples for the time of the data-collection period and thus leave out the temporal dynamics of these social interactions. This is suitable to test the hypotheses in this article, because they relate to the overall amount of social interactions. Future studies, however, could aim at understanding how depressive symptoms related to particular interaction sequences. For such research questions, time-stamped network analysis methods are a suitable framework^49–51^. Fourth, the undirected nature of the social interaction measure only allows us to draw conclusions about which interactions are more likely—and not which interactions depressed individuals seek, avoid, or terminate. Fifth, it is important to consider that effects between depression and social ties can go in both directions^9,11–14,52^: Social ties can affect individual’s levels of depressive symptoms and depressive symptoms can affect how individuals form and maintain social ties^9,11–14^. This article only focuses on the latter by showing how depressive symptoms predict social interactions. Future studies could investigate how social interactions on this weekend affected depressive symptoms later on.

Despite these limitations, our study has highlighted the strong effects that an individual’s depressive symptoms have on social interactions. We have further demonstrated that social network designs and methodologies can offer us new insights on fundamental issues of psychology and behavioral studies. We believe that an in-depth understanding of the small-scale social consequences of depressive symptoms can help to design interventions targeting the downward spiral of depression and social isolation more effectively.

## Methods

### Participants

We investigated our research questions with two independent datasets newly formed undergraduate student cohorts attending a voluntary social event on the first (sample one) and second (sample two) weekend of their studies. The data was collected in the context of the *Swiss StudentLife* study^53^. The data that is analyzed in this article and the analysis script can be downloaded from osf.io/4sj4s.

The first sample consisted of N_1_ = 73 individuals, of which 14 individuals belonged to the student organization that organized the event. The second sample consisted of *N*_2_ = 50 individuals, including 14 student organization members. Prior to the weekend, 53 (73%; Sample one) and 48 (96%; Sample two) of the participants administered an online survey that assessed friendship ties within the cohort and depressive symptoms. None of the student organization members of sample one participated in the survey. All non-responses were treated as missing data. The first sample was predominately male (37% female), whereas the second sample was mostly female (60%). The mean age of the two samples were 20.75 years (*SD* = 2.09) and 21.73 years (*SD* = 3.24), respectively. In total, there were 3,853 dyadic relations ($\frac{={N}_{1}({N}_{1}-1)}{2}=2,628$; ${N}_{2}^{dyads}=1,225$) of which 2,454 (64%) remain after listwise deletion of missing data. Hence, the sample size for our analyses should be sufficiently large.

### Procedure

In the three days prior to the weekend (Tuesday to Thursday) participants were invited to administer the online questionnaire. The study was advertised as a broad investigation about social integration and the lives of students in the first year at university.

Before the arrival at the remotely located camp house, each participant was equipped with a badge that consisted of the active Radio Frequency Identification device (RFID; see Fig. 1), which allowed us to measure their social interactions^35,36^. The badge was covered with a piece of paper with the participant’s name printed on it. Hence, the RFID badge was not visible. Participants were briefed on the badge’s functionality and purpose of application. All participants were instructed to wear the RFID badge during their time spent awake and place them on chest height. In both samples, all of the participants agreed to wear the badge throughout the weekend. During the event, study confederates checked that the participants wore the badge correctly. After an initial excitement about the badges, participants soon did not seem to notice or discuss them frequently. The events were scheduled in late September 2016 from Friday 7 pm to Sunday 8am (sample one) and in early October 2016 from Saturday 3 pm to Sunday 11 pm (sample two). During the course of the weekend, there were some organized activities (e.g., group games, lectures), but most of the time was unstructured so that participants could freely interact with each other (structured time in Sample 1 was 120 minutes, in Sample 2 45 minutes).

### Measures

#### Social interactions

During the course of the weekend, social interactions were assessed using active Radio Frequency Identification (RFID) badges. The hardware was constituted of 2.4 GHz RFID badges with realtime proximity and position tracking utilizing the Bluetooth low energy protocol. RFID badges measure proximity to other RFID badges up to 1.6 meters. Because the signal is shielded towards the back by the participant’s body, they only measure frontal face-to-face social interactions. The validity of RFID badges to measure social interactions has been shown in Elmer *et al*.^36^.

To detect the signal between two RFID badges, both badges need to be close to each other (range 1-1.5 m)^35^ and to an RFID reader. RFID readers are designed to receive signals from RFID badges that are in the range of 10 meters from a reader. Before the arrival of the participants, the camp house was equipped with 8 RFID readers so that in every room of the house and in commonly used outside areas (e.g., smoking area) signals between RFID badges could be detected. We followed the recommendations by Elmer *et al*.^36^, to enhance the validity of RFID badges by merging interactions of the same dyad if the signals are no longer than 75 seconds apart. Robustness analyses conducted on data that was not processed in that way can be found in Table S1 and Table S2 of the Supplementary Materials. More details on the RFID badges to measure face-to-face interactions can be found elsewhere^35,36^.

The dependent variable in our subsequent analyses is the duration of these social interactions. The dependent variable is in our case an adjacency matrix, in which each cell indicates how long two individuals interacted with one another throughout the whole weekend. Hence, the adjacency matrix is undirected, symmetric, and weighted.

Existing studies predominately use self-report measures to assess social interactions. Biases in self-reports of individuals with depressive symptoms might contribute to differences in their self-reported interactions, as – for instance – depressed individuals tend to view things more negatively than non-depressed^26^. With the RFID based method of social interaction measurement, we aim to overcome these biases.

#### Friendship ties

Friendship ties were measured with the items “which of your fellow students would you call friends?” (German original: “Welche Deiner Mitstudierenden würdest Du als Freunde bezeichnen?”). Below the item were 20 name generators displayed (i.e., text boxes where participants could enter the names of the individuals). An auto-complete function suggested the full names of other participants when starting to type in this text field. The nominations of that item were used to construct a binary adjacency matrix *A* where each entry *a*_*ij*_ represents the nomination of individual *j* by individual *i* (0 = no nomination, 1 = nomination).

Because our statistical method requires the independent variables to be symmetric matrices (for details see Section Statistical Analyses), we constructed a symmetrized friendship matrix indicating if at least one *i*→*j* or *j*→*i* friendship nomination was present. To explore the unique contribution of weak and strong friendship ties, two additional adjacency matrices were created in which cells indicate if the tie is (i) a mutual (strong) friendship tie (i.e., *i*→*j* and *j*→*i*) or (ii) an asymmetric (weak) friendship tie (i.e., either *i*→*j* or *j*→*i*, but not a mutual tie). These measures can be used for explorations of friendship strength^29^ and stability^54^.

#### Depressive symptoms

Depressive symptoms were measured with the German version of the Center for Epidemiologic Studies Depression Scale – Revised^55^ with 20 items on a 4-point scale ranging from 0 (occurred never or rarely) to 3 (occurred most of the time or always) reflecting how often the respective symptoms was experienced during the preceding week. Sample items are for instance “feeling depressed” or “feeling everything one does is an effort”. The depression score was computed by taking the sum of all 20 items. The total range of symptoms reflects the continuum between well-being and depression^56^. The items of this scale were highly internally consistent (Cronbach’s alpha = 0.84).

#### Big five personality traits

We conducted additional analyses to control for the effects of personality traits on social interaction tendencies. The Big Five Personality traits (openness, conscientiousness, extraversion, agreeableness, neuroticism) were measured with the 10 item version of the Big Five Inventory (BFI)^57^ where every trait is measured with two items each, rated on a 5-point Likert scale ranging from “disagree strongly” (1) to “agree strongly” (5). A sample item for neuroticism is “I see myself as someone who: is relaxed, handles stress well” (inverse coded). The internal consistency of these item varied from *α* = 0.33 (agreeableness) to *α* = 0.80 (extraversion) and the mean values were between 2.87 (neuroticism) and 3.61 (openness).

### Statistical analyses

We investigate our hypotheses using Multiple Regression Quadratic Assignment Procedures (MRQAP)^37,38^. In social network analysis, MRQAPs are considered a core method to analyze weighted networks. The MRQAP allows us to test the depression-isolation, depression-homophily, and depression-friendship hypotheses while accounting for the interdependent nature of the social network data.

There are several reasons for choosing this statistical model over other well-established statistical such as Exponential Random Graph Models (ERGMs)^58^, Stochastic Actor-Oriented Models (SAOMs)^59^, or relational event models^49,50^. First, MRQAPs allow for the analysis of weighted social networks, making it possible to analyze our social interaction adjacency matrix that constitutes of a continuous measure of how long two individuals interacted with one another. Second, statistical models that allow the modeling of time-stamped network data (such as ours), cannot model the duration of social interactions, but only the decisions to create a social interaction. Hence, using such a model would misalign the focus of the analyses with those of our hypotheses; on the interaction creation aspect but not the duration of such. Given the fluctuation in interaction signals in our data, the duration is a more reliable measure of social interactions that the creation. Third, the MRQAP method allows to make statements about effect sizes, which – in other network models – is mostly problematic. The only major disadvantage of the MRQAP method is that we had to aggregate the time-stamped data to the duration of the whole weekend, thus losing information about the order and frequency in which interactions happened.

Mathematically, a MRQAP is defined similarly to a linear regression model but with data arranged in matrices instead of vectors:

$${y}_{ij}={\beta }_{0}+\mathop{\sum }\limits_{k=1}^{m}{\beta }_{k}\left({x}_{ij}^{k}\right)+{e}_{ij}$$

where *y* is the dependent matrix and *m* is the number of independent matrices *x*^*k*^. Parameters *β*_*k*_ are coefficients and *e*_*ij*_ the error terms.

Indexes *i* and *j* represent two individuals in a given matrix. If *x*^*k*^ represents a friendship network, ${x}_{ij}^{k}$ would indicate that *i* considers *j* a friend. Similarly, *x*^*k*^ could represent the similarity between individuals with ${x}_{ij}^{k}$, for example, indicating the difference in depressive symptoms of individuals *i* and *j* (i.e., depression similarity). In principle, parameters of a MRQAPs can be interpreted like parameters of a linear regression model, as they are estimated with ordinal least squares (OLS) estimators. MRQAPs differ only in two ways from linear regression models. The first difference is that, the unit of analysis in MRQAPs is on the dyadic level. Hence, the dependent variable is an adjacency matrix of dyadic relations (i.e., *y*_*ij*_ is the time individuals *i* and *j* interacted). Also, the independent variables of a MRQAP need to be defined on a dyadic level. Examples for friendship and depression similarity are given above. The second difference to linear regression models is concerned with the independence assumption. Social network data violate the assumption of independent observations: For instance, a person A’s interactions with Person B cannot be assumed to be independent of Person A’s interactions with Person C. Because characteristics of Person A - e.g., being female - affect both interactions. For this reason, the standard errors obtained through OLS estimation cannot be used for statistical inference. MRQAPs consider the dependencies between observations in the estimation of standard errors by relying on permutation tests for statistical inference: The OLS regression results obtained with the observed adjacency matrix are compared to a large number of regression results in which the dependent matrix *y* has been permuted. According to Dekker *et al*.^38^, the Y-permuted MRQAPs are (among the MRQAP methods) the most conservative method to obtain statistical inference—others are for example permutations of the independent variables. When permuting the dependent matrix *y*, random rows and columns are swapped, while the independent variables *x* remain unaffected. This way, structural aspects of the dependent network are preserved (e.g., the outdegree distribution), while generating a distribution that assumes no association between *y* and the *x*^*k*^. Because of the permutation-based statistical inference of the MRQAP framework, no standard errors or confidence intervals of the estimates can be computed. Thus, we rely on p-values for statistical inference. However, we also report the results of a multivariate linear regression model in Table S1 of the Supplementary Materials, where confidence intervals are reported. Within the MRQAP framework, the p-value is calculated based on the percent rank of the estimate of the observed network in the distribution of estimates based on permuted networks. For instance, the percent rank of 0.99 indicates that 99 percent of the coefficient based on permuted networks are smaller or equal to the observed estimate. The probability of observing larger estimates under the null-hypothesis is thus p = 0.01 (two-sided p-value)^37,38^.

We analyze the two samples jointly and, therefore, use a *multi-group* MRQAP, in which the dependent matrices *y* of the two samples are permuted separately^38,60^. The implementation of a multi-group MRQAP function in R, is made available on the public Open Science Framework repository of this study (osf.io/4sj4s).

Because the distribution of the residuals of the MRQAP model with this dependent matrix was highly skewed (s = 4.00), the linear regression assumption of normality of errors was violated. Thus, we log-transformed the dependent matrix (skewness of residuals after transformation: s = 0.26) following standard procedure in linear regression models. In Table S1 of the Supplementary Materials we also report results based on non-transformed variables.

The independent matrices ${x}_{ij}$ in our MRQAP model represent either dyad-level aggregates of individual’s attributes (e.g., the difference in age of the two individuals) or dyadic relations (e.g., friendship nominations).

We test the *depression-isolation* hypothesis with the *depression mean* matrix, where each entry constitutes the mean depression score of both individuals *i* and *j*. The *depression-homophily* hypothesis is tested with the *depression similarity* matrix, which consists of values representing the degree of similarity in depression (${x}_{ij}=(-1)\vee {v}_{i}-{v}_{j}\vee$, were *v*_*i*_ is the depression value for individual *i*). Given that the reference category for the depression similarity effect is being identical on the depression score, the “raw” depression mean effect can be interpreted as the effect of both individuals being equally depressed. We included an interaction of these two matrices to account for differences in the importance of homophilic processes depending on the levels of depression.

To what extent depressed individuals interact with their friends (*depression-friendship hypothesis*) is tested with interactions of the depression mean and a friendship matrix. Friendship was defined when at least one of two individuals of a dyad reported a friendship tie. Whether or not friendship is mutual or asymmetric can be potentially relevant and serve as an indicator of relationship strength^29^ and stability^54^. For this reason, we conducted additional analyses in which we also consider the mutual and asymmetric friendship ties as separate independent matrices (i.e., a binary matrix indicating when both individuals nominated each other as friends and a binary matrix indicating whether or not exactly one individual of the dyad nominated the other as a friend).

Additionally, we included a dummy variable indicating whether or not the data was collected in sample two. To control for the effect of gender, we added dummy matrices as independent variables for the case of at least one female being in the interaction and for both individuals being female. Age-related effects were included with a centered age mean matrix (${x}_{ij}^{k}=\frac{({v}_{i}-\bar{v})+({v}_{j}-\bar{v})}{2}$) and an age similarity matrix (${x}_{ij}^{k}=(-1)\vee {v}_{i}-{v}_{j}\vee$, where *v*_*i*_ is the age value for individual *i*).

In the supplementary analyses, we control for the effects of the Big Five personality traits on social interactions. For this, we constructed two matrices for each trait that represent the centered mean value of *i* and *j* in the respective trait as well as their similarity in that trait.

*Dyadic isolation* is evaluated outside the MRQAP framework. For this, we computed the number of seconds that each individual spent in either a dyadic or group interaction (i.e., at least three individuals present in the social interaction). These two variables are then compared to each other with respect to an individual’s depression scores to assess the degree of dyadic isolation. To test this hypothesis, we compute the Pearson correlation between an individual’s depression score and the ratio of dyadic interactions in all social interactions. We compare this correlation to those of 5,000 permuted variables, representing the null distribution.

### Ethics approval

The study was reviewed and approved by the institutional ethics committee of ETH Zürich (approval 2016-N-27). The study was carried out in accordance with the relevant guidelines and regulations. Informed consent was obtained from all participants.

## Supplementary information

Supplementary Material.

## Data Availability

The datasets generated during and/or analysed during the current study are available in the Open Science Framework repository, osf.io/4sj4s.
