# Supplementary material for: Depressive symptoms are associated with social isolation in face-to-face interaction networks
Source: Sci Rep. 2020 Jan 29;10:1444. doi: 10.1038/s41598-020-58297-9 (PMC6989520; doi:10.1038/s41598-020-58297-9)
Supplement: Supplementary file 1 — Supplementary Material. [file 41598_2020_58297_MOESM1_ESM.docx]

Supplementary Material:

**Depressive symptoms are associated with social isolation in face-to-face interaction networks**

Timon Elmer^1^ & Christoph Stadtfeld^1^

^1^Chair of Social Networks

ETH Zürich

# **Robustness Analyses**

In the following section, we have conducted additional analyses to test the robustness of our findings. For this, we have run (1) a regular linear regression model (Table S1) and MRQAPs with (2) a non-log-transformed dependent variable (Table S1), (3) non-merged RFID data (interactions of dyads that were no longer than 75 seconds apart, have been merged as recommended by Elmer et al.^1^ for an improved validity; Table S1), (4) the two samples separately (Table S1), and (5) including measures of the Big Five personality traits (Table S2). All these analyses confirm that the main findings of the article are robust against different ways of treating the data. Only the depression similarity effect did not replicate in sample two and with a non-log-transformed dependent variable, as well as the depression isolation effect with a non-log-transformed dependent variable.

In the preregistration of this study ([www.osf.io/xce9g](http://www.osf.io/xce9g)) , we intended to investigate not only the effect of depressive symptoms on the interactions with friends, but also with acquaintances. We omitted the acquaintance network from this analysis to reduce the complexity of the story of the paper. Nevertheless, for an additional analysis we substituted the friendship network with the acquaintance network, which contained all friendship nominations. The main findings of the study remain robust and the interactions of the depression mean matrix with the acquaintance network show a similar pattern (β_mutual acquaintance * depression mean_ = −0.118, p = .007, β_asymmetric acquaintance * depression mean_ = 0.022, p = .720).

Moreover, the preregistered analysis plan was modified in two ways: First, testing the depression isolation hypothesis with a matrix, in which all cells in a row contain the depression value of the individual, was not feasible due to the undirected nature of the social interaction network. Hence, we had to aggregate the depression scores to the dyadic level and take the mean depression value. Second, the dependent variable was log-transformed because the linear regression assumption of normality of errors was violated when modeling the non-log-transformed dependent variable.

Table S1

*Results of five models using different data sources or statistical inference methods*

|  | Linear Regression | | | Non-Transformed | | Non-Merged | | Sample 1 | | Sample 2 | |
| --- | --- | --- | --- | --- | --- | --- | --- | --- | --- | --- | --- |
|  |  | 95% CI | |  |  |  |  |  |  |  |  |
|  | Est. | lower | upper | Est. | p | Est. | p | Est. | p | Est. | p |
| intercept | 2.504*** | 1.598 | 2.534 | -9.630* | .018 | 2.066* | .022 | 2.926*** | <.001 | 3.163$\dagger$ | .062 |
| sample two | 0.806*** | 0.532 | 0.842 | 10.759 | .141 | 0.687 | .288 | - | - |  |  |
| at least one female | -0.095 | -0.239 | 0.040 | -1.386 | .312 | -0.100$\dagger$ | .078 | -0.194* | .024 | 0.024 | .435 |
| both female | -0.148 | -0.268 | 0.018 | 0.660 | .423 | -0.125* | .046 | 0.02 | .433 | -0.278** | .003 |
| age mean (centered) | 0.065* | 0.008 | 0.108 | 0.259 | .395 | 0.058* | .011 | 0.008 | .445 | 0.116** | .002 |
| age similarity | 0.042* | 0.011 | 0.073 | 1.200* | .046 | 0.042** | .007 | 0.033 | .124 | 0.050* | .016 |
| one student organization^1^ | -0.028 | -0.315 | 0.477 | 32.489** | .004 | 0.081 | .338 | - | - | -0.189 | .211 |
| same student status^1^ | 0.269 | -0.059 | 0.719 | 38.114** | .001 | 0.330$\dagger$ | .058 | - | - | 0.209 | .176 |
| being friends | 2.128*** | 1.628 | 2.445 | 104.378*** | <.001 | 2.036*** | <.001 | 2.265*** | <.001 | 1.974*** | <.001 |
| depression mean | -0.059*** | -0.068 | -0.027 | -0.474 | .160 | -0.047*** | <.001 | -0.064*** | .001 | -0.042** | .003 |
| depression similarity | 0.047** | 0.008 | 0.067 | 0.557 | .213 | 0.038** | .008 | 0.069* | .018 | 0.024 | .142 |
| depression mean * depression similarity | -0.004*** | -0.005 | -0.001 | -0.016 | .344 | -0.003** | .002 | -0.004* | .041 | -0.002* | .036 |
| depression mean * being friends | -0.012 | -0.052 | 0.041 | 1.958$\dagger$ | .053 | -0.006 | .404 | 0.036 | .270 | -0.013 | .349 |
| R^2^ | .123 |  |  | .168 |  | .128 |  | .068 |  | .125 |  |
| Adj. R^2^ | .119 |  |  | .164 |  | .124 |  | .062 |  | .116 |  |

*Note.* $N_{sample1+2}=2,454$, (Multigroup) MRQAPs with 5,000 Y-permuted samples. CI = confidence interval, $\dagger$ *p* $<$ .10, * *p* $<$ .05, ** *p* $<$ .01, *** *p* $<$ .001 (two-sided p-values). Non-Merged = the interactions were not merged according to the recommendations of Elmer et al.^1^, Non-Transformed = the dependent variable (interaction durations) has not been log-transformed. ^1^no estimate of sample one because of missing depression scores of student organization members.

**Multi-group MRQAP controlling for personality traits**

To take into account that an individual’s personality can affect the formation of social interactions, we conducted a further multi-group MRQAP analysis including effects for the Big Five personality traits. The results of this multi-group MRQAP analysis including personality traits can be found in Table S2. The findings of hypothesis one and two were robust in size and significance when including personality traits in the model.

Table S2

*Multi-group MRQAP results on log transformed interaction durations of dyads including personality traits*

|  | Est. | p |
| --- | --- | --- |
| intercept | 3.363*** | <.001 |
| sample two | 0.774 | .242 |
| at least one female | -0.431*** | <.001 |
| both female | -0.221** | .008 |
| age mean (centered) | 0.078** | .006 |
| age similarity | -0.041* | .016 |
| one student organization | -0.326$\dagger$ | .080 |
| same student status | 0.132 | .278 |
| being friends | 2.079*** | <.001 |
| openness mean (centered) | -0.135* | .015 |
| openness similarity | 0.087* | .028 |
| conscientiousness mean (centered) | 0.369*** | <.001 |
| conscientiousness similarity | -0.046 | .180 |
| extraversion mean (centered) | -0.126* | .017 |
| extraversion similarity | 0.066$\dagger$ | .076 |
| agreeableness mean (centered) | -0.278*** | <.001 |
| agreeableness similarity | -0.106* | .026 |
| neuroticism mean (centered) | 0.202*** | <.001 |
| neuroticism similarity | 0.001 | .477 |
| depression mean | -0.081*** | <.001 |
| depression similarity | 0.051** | .003 |
| depression mean * depression similarity | -0.004*** | <.001 |
| depression mean * being friends | -0.010 | .362 |
| R^2^ | .143 |  |
| Adj. R^2^ | .134 |  |

*Note.* $N=2,454$, Multigroup MRQAP with 5,000 Y-permuted samples. $\dagger$ *p* $<$ .10, * *p* $<$ .05, ** *p* $<$ .01, *** *p* $<$ .001 (two-sided p-values).

**The Role of Friendship Strength**

To explore the role of friendship strength when testing the *depression-friendship hypothesis* we conduced additional analyses in which we differentiate between asymmetric and mutual friendship ties. We generally assume that mutual friendship ties (i.e., person A is nominating person B and person B is nominating person A) are stronger than asymmetric friendship ties (i.e., only either person A nominates person B or person B nominates person A)^3^. Table S3 shows the results of the MRQAP model including separate effects for mutual and asymmetric friendship ties. These results indicate that depressive symptoms are associated with spending less time with strong (i.e., mutual) friends. We find no effect of the interaction of asymmetric friendship ties with the depression mean matrix when predicting social interactions. When conducing the above-mentioned robustness checks (see Section Robustness Analyses) with this model specification, we consistently find support that depressive symptoms are associated with interacting less with strong friends (model results are not reported here due to space restrictions). Also, the findings of the depression-isolation and the depression-homophily hypotheses remain robust with this alternative model specification.

Table S3
*Multi-group MRQAP results on log transformed interaction durations of dyads*

|  | Est. | p |
| --- | --- | --- |
| intercept | 2.501** | .006 |
| sample two | 0.794 | .268 |
| at least one female | -0.099 | .106 |
| both female | -0.152* | .038 |
| age mean (centered) | 0.062* | .019 |
| age similarity | 0.041* | .012 |
| one student organization | -0.012 | .487 |
| same student status | 0.276 | .108 |
| being mutual friends | 3.102*** | <.001 |
| being asymmetric friends | 1.224* | .019 |
| depression mean | -0.059*** | <.001 |
| depression similarity | 0.047** | .002 |
| depression mean * depression similarity | -0.004*** | <.001 |
| depression mean * being mutual friends | -0.084* | .029 |
| depression mean * being asymmetric friends | 0.069 | .095 |
| R^2^ | .125 |  |
| Adj. R^2^ | .120 |  |

*Note.* $N_{sample1+2}=2,454$, Multigroup MRQAP with 5,000 Y-permuted samples. $\dagger$ *p* $<$ .10, * *p* $<$ .05, ** *p* $<$ .01, *** *p* $<$ .001 (one-sided p-values).

**Computation of the Selection Table**

In Figure 3 of the main text we show the likelihood of interactions between two individuals $i$ and $j$ based on their depressive symptoms (i.e., $v_{i}$ and $v_{j}$). Here we report how we computed these values.

Considering that everything else is the reference category (e.g., male dyad interaction, non-friends), we get the following formula for the estimate of interaction duration of the dyad ${\overset{̂}{y}}_{ij}$ based on depression mean and depression similarity:

$${\overset{̂}{y}}_{ij}=\exp((\beta_{0}+\beta_{1}\frac{v_{i}+v_{j}}{2}+\beta_{2}(-1)|v_{i}-v_{j}|+\beta_{3}\frac{v_{i}+v_{j}}{2}(-1)|v_{i}-v_{j}|)$$

where $\beta_{0,1,2,3}$ denote the estimates for the intercept, depression mean, depression similarity and the interaction of the depression mean and the depression similarity matrix. Variables $v_{i}$ and $v_{j}$ are the depression scores of actor $i$ and $j$. Taking the respective estimates from Table 2 of the main text for values of the observed depression scores we obtained a table of estimated ${\overset{̂}{y}}_{ij}$. If we consider two example dyads, one where individuals report a depression score of $v_{i}=$ 4 and $v_{j}=$ 6, and the other where values of $v_{i}=$ 4 and $v_{k}=$ 16 were reported, we would get estimates of ${\overset{̂}{y}}_{ij}=$ 8.61 seconds per hour and ${\overset{̂}{y}}_{ik}=$ 6.00 seconds per hour, respectively. Using the above introduced equation we then computed ${\overset{̂}{y}}_{ij}$ for each combination of the observerd values of depressive symptoms to construct the table/heatmap reported Figure 3 of the main text.

**References**

1. Elmer, T., Chaitanya, K., Purwar, P. & Stadtfeld, C. The validity of RFID badges measuring face-to-face interactions. *Behav. Res. Methods* 1–19 (2019). doi:10.3758/s13428-018-1180-y

2. Rammstedt, B. & John, O. P. Measuring personality in one minute or less: A 10-item short version of the Big Five Inventory in English and German. *J. Res. Pers.* **41,** 203–212 (2007).

3. Friedkin, N. E. A Guttman Scale for the Strength of an Interpersonal Tie. *Soc. Networks* **12,** 239–252 (1990).
